# Performance of Large Language Models (ChatGPT and Gemini Advanced) in Gastrointestinal Pathology and Clinical Review of Applications in Gastroenterology

**DOI:** 10.7759/cureus.81618

**Published:** 2025-04-02

**Authors:** Swachi Jain, Baidarbhi Chakraborty, Ashish Agarwal, Rashi Sharma

**Affiliations:** 1 Pathology and Laboratory Medicine, Icahn School of Medicine at Mount Sinai, New York, USA; 2 Pathology, St. Clare Hospital, Denville, USA; 3 Data Science, Amazon, Seattle, USA; 4 Pathology and Laboratory Medicine, Medanta-The Medicity, Gurgaon, IND

**Keywords:** accuracy, artificial intelligence, chatgpt, diagnosis, gastrointestinal tract, gemini, general gastroenterology, healthcare, large language models, pathology

## Abstract

Introduction

Artificial intelligence (AI) chatbots have been widely tested in their performance on various examinations, with limited data on their performance in clinical scenarios. The role of Chat Generative Pre-Trained Transformer (ChatGPT) (OpenAI, San Francisco, California, United States) and Gemini Advanced (Google LLC, Mountain View, California, United States) in multiple aspects of gastroenterology including answering patient questions, providing medical advice, and as tools to potentially assist healthcare providers has shown some promise, though associated with many limitations. We aimed to study the performance of ChatGPT-4.0, ChatGPT-3.5, and Gemini Advanced across 20 clinicopathologic scenarios in the unexplored realm of gastrointestinal pathology.

Materials and methods

Twenty clinicopathological scenarios in gastrointestinal pathology were provided to these three large language models. Two fellowship-trained pathologists independently assessed their responses, evaluating both the diagnostic accuracy and confidence of the models. The results were then compared using the chi-squared test. The study also evaluated each model's ability in four key areas, namely, (1) ability to provide differential diagnoses, (2) interpretation of immunohistochemical stains, (3) ability to deliver a concise final diagnosis, (4) and explanation provided for the thought process, using a five-point scoring system. The mean, median score±standard deviation (SD), and interquartile ranges were calculated. A comparative analysis of these four parameters across ChatGPT-4.0, ChatGPT-3.5, and Gemini Advanced was conducted using the Mann-Whitney U test. A p-value of <0.05 was considered statistically significant. Other parameters evaluated were the ability to provide a tumor, node, and metastasis (TNM) stage and the incidence of pseudo-references "hallucinations" while citing reference material.

Results

Gemini Advanced (diagnostic accuracy: p=0.01; providing differential diagnosis: p=0.03) and ChatGPT-4.0 (interpretation of immunohistochemistry (IHC) stains: p=0.001; providing differential diagnosis: p=0.002) performed significantly better in certain realms than ChatGPT-3.5, indicating continuously improving data training sets. However, the mean performances of ChatGPT-4.0 and Gemini Advanced ranged between 3.0 and 3.7 and were at best classified as average. None of the models could provide the accurate TNM staging for these clinical scenarios, with 25-50% citing references that do not exist (hallucinations).

Conclusion

This study indicated that though these models are evolving, they need human supervision and definite improvements before being used in clinical medicine. This is the first study of its kind in gastrointestinal pathology to the best of our knowledge.

## Introduction

Artificial intelligence (AI) models are computer programs designed for pattern recognition through training on extensive datasets. A subset of AI, known as generative AI, utilizes deep learning techniques to autonomously produce diverse forms of novel content, including text, images, audio, code, and data [1]. These generative AI models can respond to queries, interpret information closely resembling human output, continuously learn from ongoing data inputs, and progressively enhance their capabilities [1].

In this explosion of AI, the advent of large language models (LLMs) led to the development of various applications, such as Chat Generative Pre-Trained Transformer (ChatGPT) by OpenAI (San Francisco, California, United States), Gemini by Google LLC (Mountain View, California, United States), and Claude by Anthropic (San Francisco, California, United States) [2]. The growth of ChatGPT has been phenomenal, with 180.5 million users and the website openai.com receiving approximately 3.66 billion visits in November 2024 [3]. Google's Gemini has 275 million monthly visitors as of January 2025 and is available in 230 countries [4]. These models are continuously evolving with ChatGPT integrating with DALL-E and allowing the interpretation and generation of images in October 2023 and Gemini Advanced following suit in February 2024 [5,6].

The application of ChatGPT in healthcare is being extensively studied and has shown potential in providing information about medicine, providing medical advice, and interacting with patients. There have been few studies evaluating the role of ChatGPT in various aspects of gastroenterology. Lahat et al. evaluated the utility of ChatGPT in answering patient questions in real-life situations related to gastroenterology [7], while Lim et al. studied the answers provided by ChatGPT to patients regarding endoscopy queries [8]. In other studies, even though there were limitations, ChatGPT showed potential in assisting healthcare providers with the treatment of patients with gastroesophageal reflux disease (GERD) and the management of patients following colonoscopy [9,10]. ChatGPT was unsuccessful in passing the gastroenterology board examinations [11].

As pathology plays an integral part in the diagnosis of gastrointestinal (GI) diseases, we aimed to study AI models in the unexplored realm of GI pathology. Initial studies showed the use in pathology is still in the primitive phase with potential in (1) generating differential diagnoses (D/D) and recommending the next steps for testing, (2) modifying responses based on the input of test results, (3) interpreting diagnostic stains in histopathology, and (4) identifying unusual subtypes of pathologies. However, there are major limitations, including the fabrication of references provided, incorrect information in a confident tone (hallucinations), and challenges inherent to the largely image-based nature of pathology [12,13].

Our research aimed to evaluate the performance of ChatGPT-4.0, ChatGPT-3.5, and Gemini Advanced across 20 clinicopathologic scenarios in GI pathology. The study focused on assessing each model's ability to provide differential diagnoses, interpret immunohistochemical (IHC) stains, and provide a concise final diagnosis. Additionally, we compared the outputs from the three chatbots to determine their relative efficiencies. There are limited studies comparing the efficiency of ChatGPT-4.0, ChatGPT-3.5, and Gemini Advanced, especially in pathology, and ours is the first study of this kind in GI pathology to the best of our knowledge.

## Materials and methods

The performance of ChatGPT-4.0, ChatGPT-3.5, and Gemini Advanced was evaluated using 20 potential real-life clinical scenarios (Appendix A) faced by pathologists in their practice of neoplastic GI pathology (pancreas=4, stomach=4, colorectum=6, appendix=2, esophagus=2, peritoneum=1, metastases=1). The questions were designed to cover a wide range of topics to reflect some typical and some rare entities encountered on the GI service.

The scenarios and the top three D/D provided are recorded in Table 1.

**Table 1:** D/D (top 3) and final diagnosis provided by ChatGPT-4.0, ChatGPT-3.5, and Gemini Advanced in the study D/D: differential diagnosis; WD PanNET: well-differentiated pancreatic neuroendocrine tumor; PanNET: pancreatic neuroendocrine tumor; ACC: acinar cell carcinoma; SPN: solid pseudopapillary neoplasm; PDAC: pancreatic ductal adenocarcinoma; IPMN: intraductal papillary mucinous neoplasm; MCN: mucinous cystic neoplasm; GCT: granular cell tumor; SCC: squamous cell carcinoma; GIST: gastrointestinal stromal tumor; NEC: neuroendocrine carcinoma; NET: neuroendocrine tumor; MALT: mucosa-assisted lymphoid tissue; NHL: non-Hodgkin lymphoma; EMPD: extramammary Paget disease; BCC: basal cell carcinoma; AIN: anal intraepithelial neoplasia; CRC: colorectal carcinoma; PPC: primary peritoneal carcinoma; LAMN: low-grade appendiceal mucinous neoplasm; HSIL: high-grade squamous intraepithelial lesion; TA: tubular adenoma; TVA: tubulo-villous adenoma; IFP: inflammatory fibroid polyp; SFT: solitary fibrous tumor; IMT: inflammatory myofibroblastic tumor; HCC: hepatocellular carcinoma; RCC: renal cell carcinoma; N/A: no D/D provided

Case no.	Case diagnosis	ChatGPT-4.0 top 3 D/D	ChatGPT-4.0 final diagnosis	ChatGPT-3.5 top 3 D/D	ChatGPT-3.5 final diagnosis	Gemini Advanced top 3 D/D	Gemini Advanced final diagnosis
1	WD PanNET grade 1	PanNET, solid pseudopapillary tumor, ACC	PanNET grade 1	PanNET, SPN, ACC	WD PanNET	PDAC, PanNET, IPMN	WD PanNET
2	SPN	SPN, PanNET, ACC	SPN	PanNET, SPN, PDAC	SPN	PanNET, IPMN, ACC	SPN
3	PDAC	N/A	PDAC	N/A	PDAC	N/A	PDAC
4	MCN	MCN, serous cystadenoma, IPMN	MCN with low-grade dysplasia	Serous cystadenoma, MCN, SPN	MCN	MCN, IPMN, mature cystic teratoma	MCN
5	GCT	GCT, schwannoma, leiomyoma	GCT	SCC, adenocarcinoma, leiomyoma	Leiomyoma	Oncocytoma, SCC with granular cell change, GCT	GCT
6	Colonic adenocarcinoma	N/A	Adenocarcinoma	N/A	Colorectal adenocarcinoma	N/A	Colorectal adenocarcinoma
7	Gastric signet ring cell carcinoma	Signet ring cell carcinoma, poorly differentiated adenocarcinoma, diffuse-type gastric cancer	Gastric signet ring cell carcinoma	Signet ring cell carcinoma, diffuse-type gastric cancer, gastric lymphoma	Gastric signet ring cell carcinoma	Signet ring cell carcinoma, lobular breast carcinoma, metastatic adenocarcinoma with signet ring cell features	Gastric signet ring cell carcinoma
8	Spindle cell GIST, low risk of disease progression	GIST, leiomyoma, schwannoma	GIST	GIST, leiomyoma/leiomyosarcoma, schwannoma	GIST, spindle cell variant	GIST, leiomyoma/leiomyosarcoma, schwannoma	GIST
9	Esophageal SCC, poorly differentiated	SCC, adenocarcinoma, small cell carcinoma	SCC	SCC, adenocarcinoma, leiomyoma	SCC	SCC, poorly differentiated adenocarcinoma, metastatic carcinoma	SCC
10	Large cell NEC, rectum	Adenocarcinoma, carcinoid tumor, GIST	Large cell NEC	Adenocarcinoma, rectal NET, rectal small cell carcinoma	Rectal small cell carcinoma	Poorly differentiated adenocarcinoma, SCC of the rectum, NEC (small or large cell type)	Large cell NEC
11	MALT lymphoma of the stomach	MALT lymphoma, reactive lymphoid hyperplasia, other NHL	Gastric MALT lymphoma	MALT lymphoma, gastric carcinoma with lymphoid stroma, *Helicobacter pylori*-associated gastritis	MALT lymphoma	MALT lymphoma, *Helicobacter pylori* gastritis with severe reactive changes, lymphocytic gastritis	MALT lymphoma with *Helicobacter pylori* infection
12	Primary EMPD	EMPD, SCC, BCC	EMPD	SCC, AIN, condyloma acuminatum	EMPD	EMPD, SCC, anal adenocarcinoma	EMPD
13	Malignant melanoma, rectum	Malignant melanoma, poorly differentiated carcinoma, lymphoma	Malignant melanoma	Adenocarcinoma, GIST, infectious colitis	Malignant melanoma	Adenocarcinoma, NET, large cell lymphoma	Malignant melanoma, likely mucosal melanoma
14	Peritoneal malignant mesothelioma, epithelioid type	Metastatic CRC, PPC, ovarian carcinoma	Mesothelioma	Metastatic CRC, PPC, ovarian carcinoma	Diffuse, malignant mesothelioma, epithelioid type	Metastatic CRC, PPC, ovarian carcinoma	Peritoneal mesothelioma
15	Goblet cell adenocarcinoma of the appendix, low grade	LAMN, mucinous adenocarcinoma, goblet cell carcinoid	Goblet cell carcinoid	LAMN, adenocarcinoma, well-differentiated adenoma with focal atypia	LAMN	LAMN, mucinous adenocarcinoma, mucinous adenoma	NET, grade 1
16	Secondary Paget disease	EMPD, SCC, BCC	Metastatic CRC	SCC, EMPD with invasive carcinoma, AIN/HSIL	EMPD with invasive carcinoma	Adenocarcinoma, EMPD, Bowen's disease	Rectal adenocarcinoma
17	TA with high-grade dysplasia	N/A	Villous or TVA with high-grade dysplasia	N/A	TA	N/A	TVA with high-grade dysplasia
18	IFP	GIST, schwannoma, SFT	SFT	GIST, leiomyoma, IMT	Leiomyosarcoma	GIST, IFP, eosinophilic gastritis	IFP
19	Metastatic NET involving the liver	Metastatic carcinoma, NET, HCC	Metastatic NET	HCC, metastatic adenocarcinoma, cholangiocarcinoma	HCC	Metastatic NET, well-differentiated HCC, metastatic RCC	Metastatic well-differentiated NET, grade 1
20	LAMN	LAMN, mucinous adenocarcinoma, serrated polyp	LAMN	Mucinous adenocarcinoma, appendiceal adenoma, acute appendicitis with mucinous change	LAMN	LAMN, serrated polyp with dysplasia, mucosal hyperplasia	LAMN

**Figure 1:**
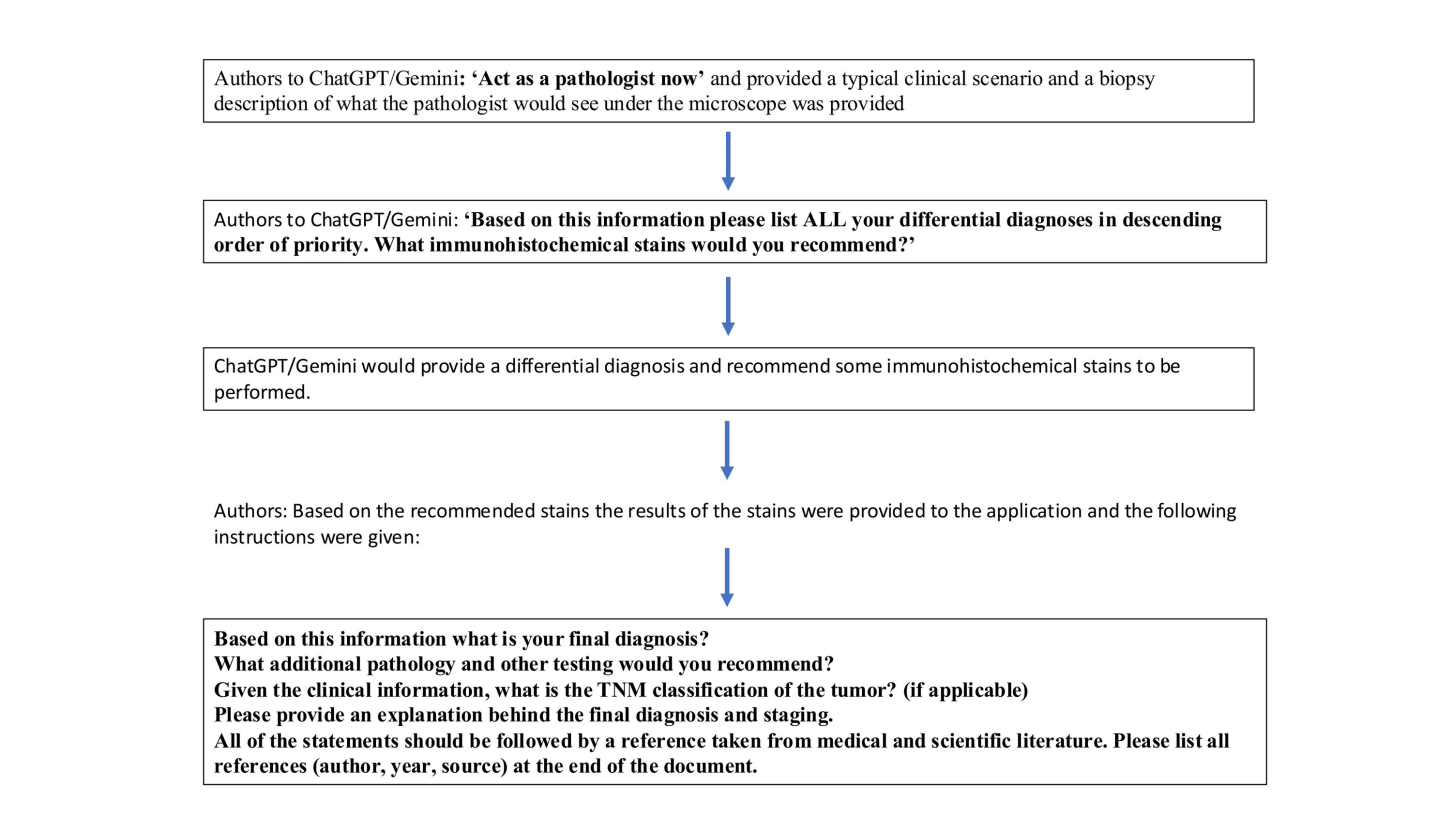
The sequence of prompts provided to ChatGPT and Gemini for this study ChatGPT: Chat Generative Pre-Trained Transformer; TNM: tumor, node, and metastasis

New tabs were used for all cases to prevent memory retention bias. The questions were put into the three AI applications (ChatGPT-4.0, ChatGPT-3.5, and Gemini Advanced). The detailed prompts are provided in Figure 1. 

The answers provided by the three AI models were evaluated independently by two GI pathology fellowship-trained pathologists, with the sample screenshots of answers shown in Appendix B. The final diagnosis was graded as correct or incorrect and was used to measure the accuracy of the applications. The confidence in their diagnosis was evaluated by assessing the percentage of answers in the first three D/D.

The pathologists assessed four additional parameters: (1) the ability to provide a D/D, (2) the recommendation and interpretation of IHC stains, (3) the ability to deliver a concise final diagnosis (similar to what a pathologist would report in the top line), and (4) the quality and clarity of the explanation provided. These parameters were scored on a scale of 1 through 5 as shown in Table 2 below.

**Table 2:** The five-point scoring system used for evaluating the responses to the clinicopathologic scenarios

Scoring system	
Score 5	Highly accurate with no errors and based on current knowledge and practice
Score 4	Moderately accurate, with minor lapses
Score 3	Vaguely accurate, few errors, and need medical supervision
Score 2	Hardly accurate, leaving a lot to be desired
Score 1	Incorrect

In nine cases, pertinent information to provide a tumor, node, and metastasis (TNM) staging was provided in the clinical vignette, and the AI models were asked to give the TNM staging. The references provided were evaluated to check for accuracy.

Statistical analysis

The statistical analysis was performed using Microsoft Excel 2019 (Microsoft Corporation, Redmond, Washington, United States) and Python 3.5 (Python Software Foundation, Wilmington, North Carolina, United States). The diagnostic accuracy for all three groups was compared using the chi-squared test. The mean, median score±standard deviation (SD), and interquartile ranges were calculated for each category. A comparison of the parameters on ChatGPT-4.0 vs. ChatGPT-3.5 vs. Gemini Advanced was performed using the Mann-Whitney U test. A p-value of <0.05 was considered statistically significant.

## Results

The results were assessed on various competencies of the LLMs and are summarized in Table 3 and Table 4.

**Table 3:** Diagnostic accuracy, confidence values, and comparison of various parameters in ChatGPT-4.0, ChatGPT-3.5, and Gemini Advanced D/D: differential diagnoses; IQR: interquartile range; SD: standard deviation; TNM: tumor, node, and metastasis

	ChatGPT-4.0	ChatGPT-3.5	Gemini Advanced
Diagnostic accuracy and confidence values			
Diagnostic accuracy (n=20, %)	N=18 (90%)	N=13 (65%)	N=19 (95%)
Confidence value (diagnosis in the first 3 D/D, n=17, %)	N=14 (82.35%)	N=13 (76.75%)	N=11 (64.70%)
Providing a differential diagnosis (n=17)			
Average score±SD	3.70±0.45	2.76±0.9	3.08±0.86
Median (IQR)	4 (3.5-4.0)	2.5 (2.0-3.5)	3 (2.5-4.0)
Interpretation of immunohistochemical stains			
Average score±SD	3.45±0.53	2.67±0.84	3.17±0.71
Median (IQR)	3.5 (3.375-4.0)	3 (2.0-3.125)	3 (2.5-4.0)
Concise final diagnosis (n=20)			
Average score±SD	3.5±0.91	2.7±1.38	3.57±0.72
Median (IQR)	4 (3.5-4.0)	3.25 (1.0-4.0)	3 (4.0-4.0)
Quality of explanation			
Average score±SD	3.33±0.40 (n=18)	2.84±0.66 (n=13)	3.26±0.65 (n=19)
Median (IQR)	3.5 (3.0-3.5)	3 (2.5-3.0)	3 (3.0-3.75)
TNM staging			
Accurate staging	None	None	None
References			
Total provided	68	91	67
Correct references	38	46	49
Hallucination (%)	44.12%	49.95%	26.8%

**Table 4:** Comparative statistical analysis of ChatGPT-4.0, ChatGPT-3.5, and Gemini Advanced Diagnostic accuracy was calculated using the chi-squared test. The remaining statistical analysis was performed using the Mann-Whitney U test, and a p-value of <0.05 is considered statistically significant.

	ChatGPT-4.0 vs. ChatGPT-3.5	Gemini Advanced vs. ChatGPT-3.5	ChatGPT-4.0 vs. Gemini Advanced
Diagnostic accuracy (n=20)			
Chi-squared value	3.58	5.62	0.36
P-value	0.36	0.01	0.54
Providing a differential diagnosis (n=17)			
U-value	231.0	204.5	171.0
P-value	0.002	0.03	0.36
Interpretation of immunohistochemical stains (n=20)			
U-value	512.0	419.0	245.5
P-value	0.001	0.08	0.13
Concise final diagnosis (n=20)			
U-value	252.5	143.5	202.5
P-value	0.13	0.099	0.95

The statistical analysis was performed using the chi-squared test and Mann-Whitney U test, as shown in Table 4.

Diagnostic accuracy

Of the 20 cases, Gemini Advanced (19/20; 95%; p=0.01) performed significantly better, and ChatGPT-4.0 (18/20; 90%; p=0.05) almost reached statistical significance compared to ChatGPT-3.5 (Table 4).

Confidence values in providing a diagnosis

Seventeen cases needed a D/D, and ChatGPT-4.0 provided the correct answer in the first three D/D in 14 cases (82.35%), Gemini Advanced in 13 cases (76.47%), and ChatGPT-3.5 in 11 cases (64.70%), as depicted in Table 3.

In cases where the correct diagnosis was not in the top three D/D, ChatGPT-4.0 could reach the correct diagnosis in two of three cases (cases 10 and 14), Gemini Advanced in three of four cases (cases 2, 13, and 14), and ChatGPT-3.5 in two of six cases (cases 13 and 14) by evaluating the IHC results provided, following human prompts and reconsidering its D/D.

Providing a D/D

**Figure 2:**
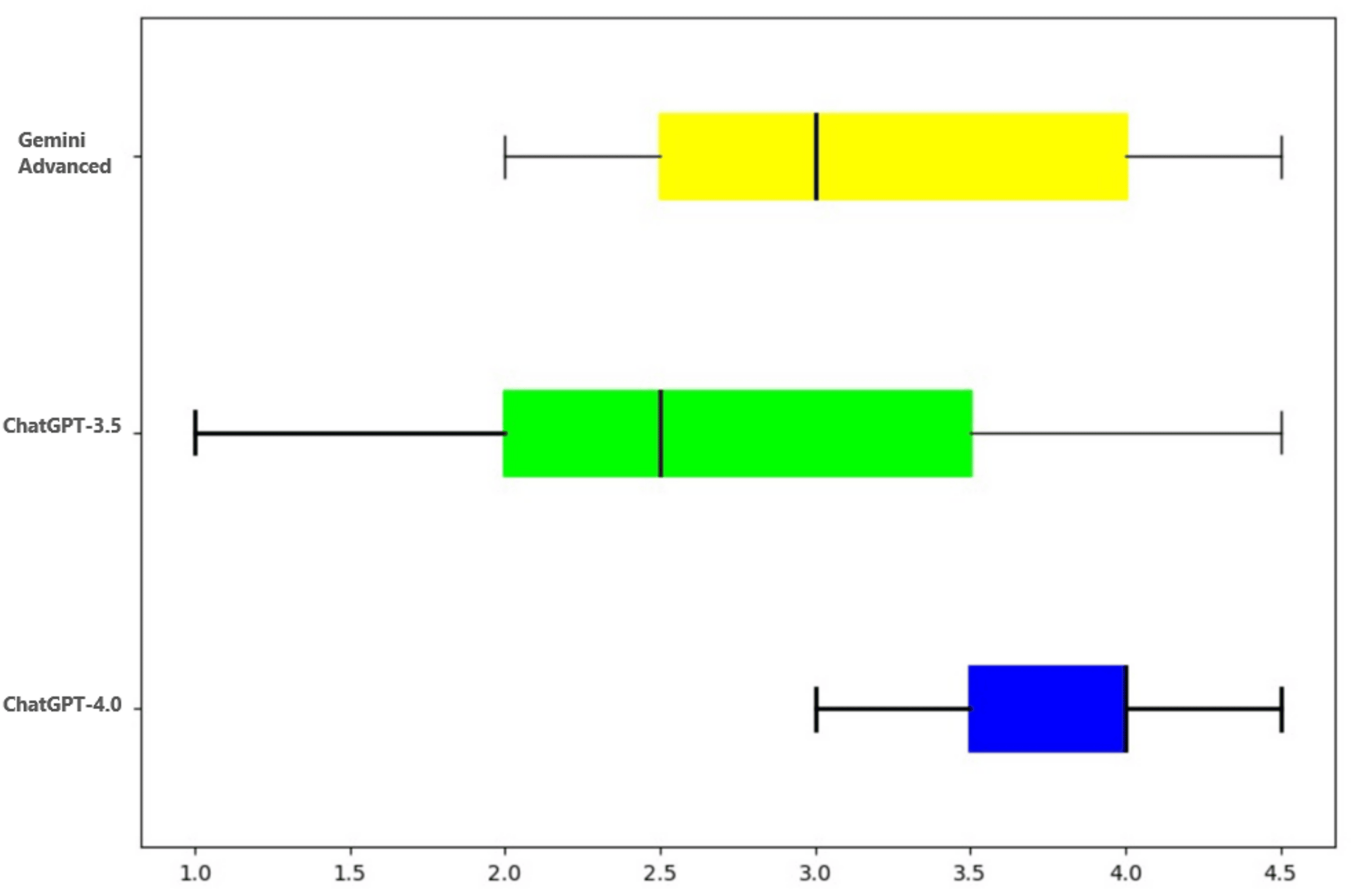
Graphical representation of scores assigned to different LLMs based on their ability to generate a differential diagnosis LLMs: large language models

The first part of the prompt was to examine the ability of the AI models to "think and reason" in a given clinical scenario by providing a D/D. Three of the 20 cases did not require a D/D. These were analyzed by two fellowship-trained pathologists, and the results are seen in Table 3 and Figure 2.

ChatGPT-4.0 and Gemini Advanced performed significantly better than ChatGPT-3.5 (p=0.002 and p=0.03, respectively) as depicted in Table 4.

Interpretation of IHC stains

**Figure 3:**
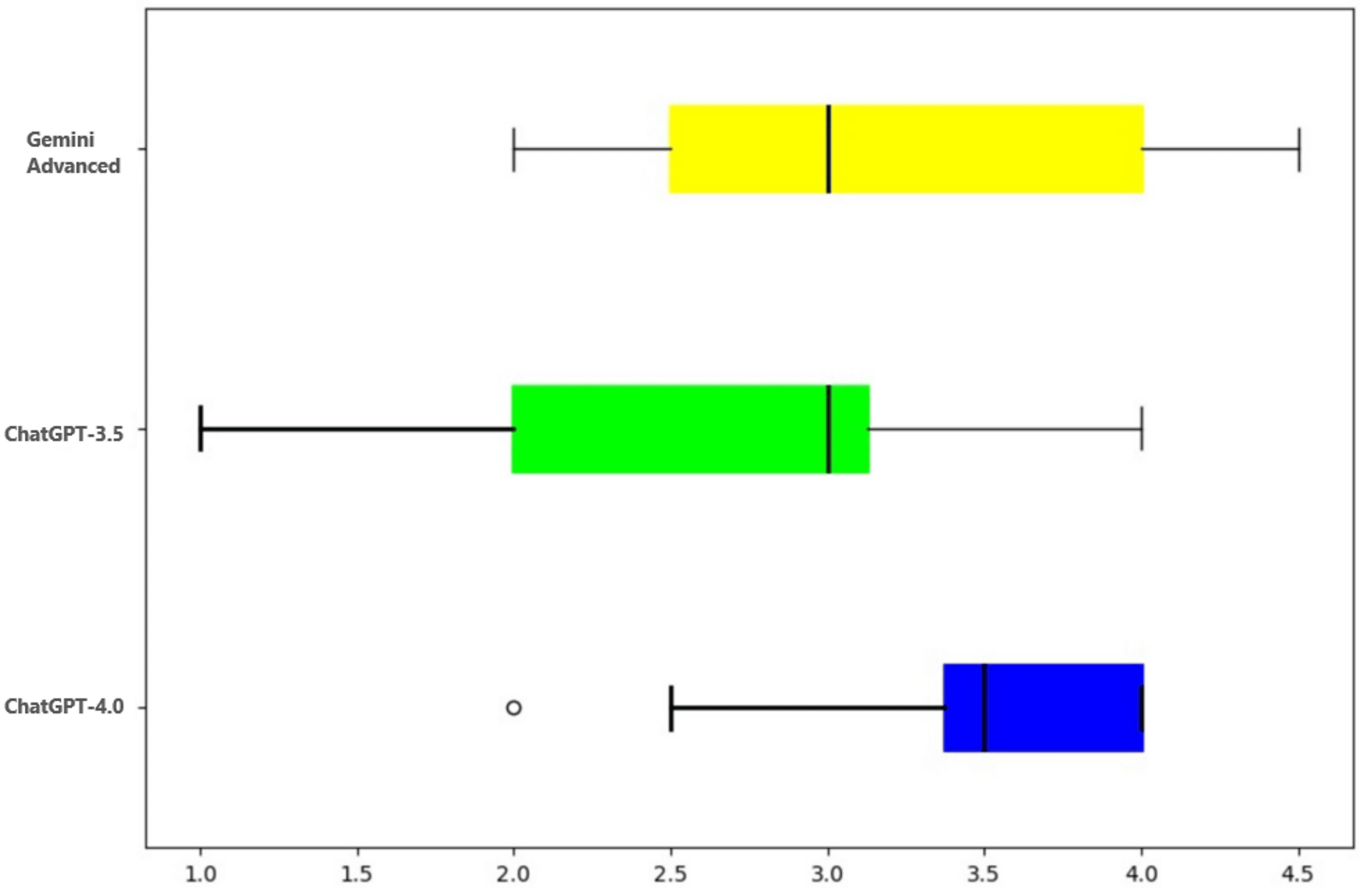
Graphical representation of scores assigned to different LLMs on their ability to recommend and interpret immunohistochemical stains LLMs: large language models

The interpretation of IHC stains was evaluated, and it was seen that ChatGPT-4.0 performed significantly better (p=0.001), followed by Gemini Advanced and ChatGPT-3.5, as depicted in Table 3, Table 4, and Figure 3.

Some interesting points were noted. ChatGPT-4.0 identified cytoplasmic staining of beta-catenin in the question in case 2 and explained that solid pseudopapillary neoplasm (SPN) of the pancreas should exhibit nuclear staining for the same. However, there were errors, e.g., in the case of pancreatic ductal adenocarcinoma (case 3), it pointed out that "CDX2 is typically negative in pancreatic ductal adenocarcinoma, while positive in metastatic colorectal adenocarcinomas to pancreas" and recommended "MUC stains as MUC1 is typically positive in pancreatic ductal adenocarcinoma, while MUC2 is indicative of intraductal papillary mucinous neoplasm (IPMN) or mucinous cystic neoplasm (MCN) of the pancreas" which are incorrect statements. 

Concise final diagnosis

**Figure 4:**
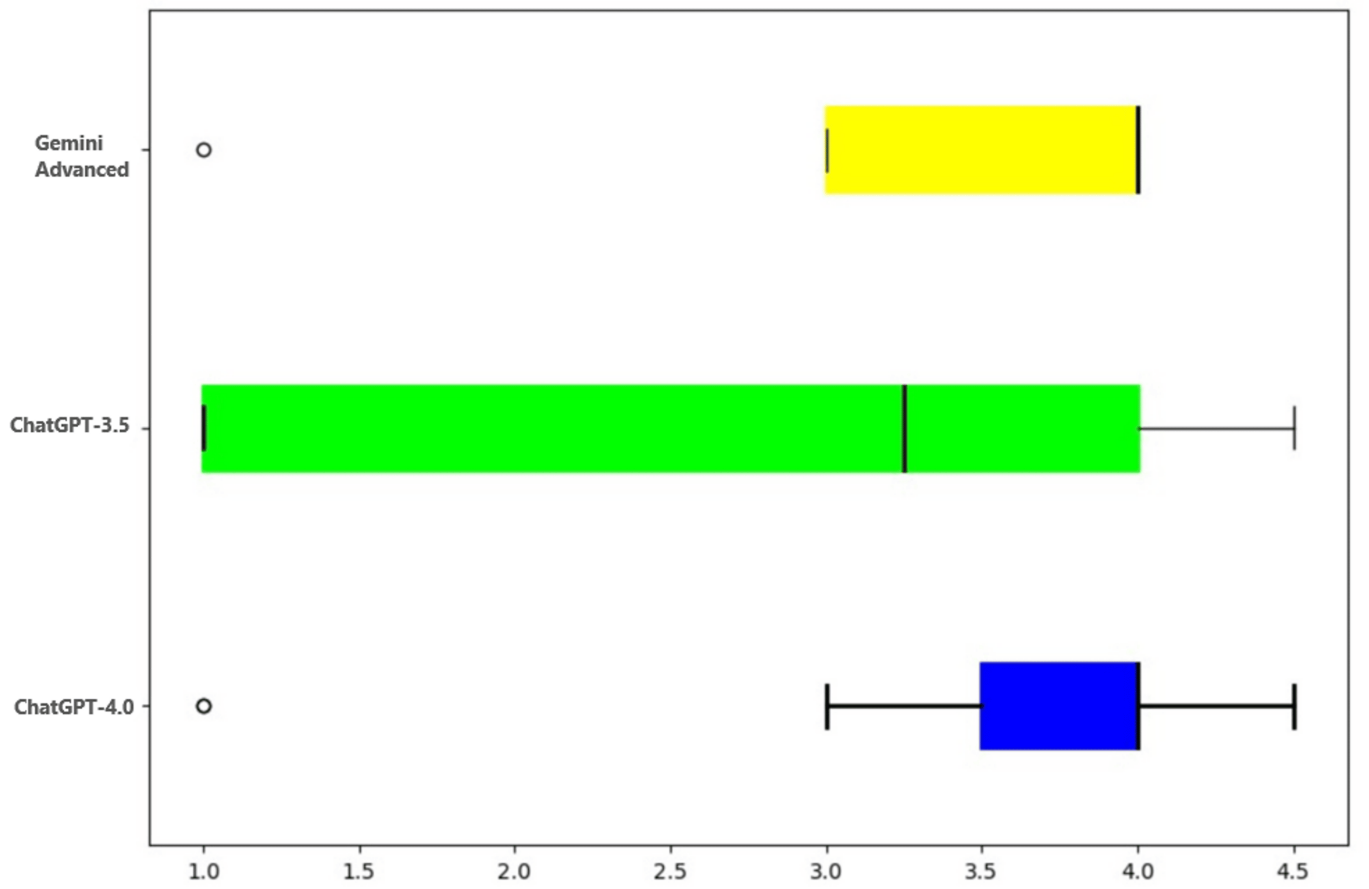
Graphical representation of scores assigned to different AI chatboxes on their ability to deliver a concise final diagnosis AI: artificial intelligence

The ability of the models to give a concise final diagnosis (akin to the top-line diagnosis at the final signout) was scored. ChatGPT-4.0 and Gemini Advanced fared similarly with ChatGPT-3.5 showing the lowest performance, as shown in Table 3, Table 4, and Figure 4.

None of the LLMs commented on additional features like grade or differentiation that are usually reported in the main diagnosis.

Quality of explanation provided

Explanations were scored based on comprehensibility and accuracy. Explanations for incorrect answers were excluded. ChatGPT-4.0 and Gemini Advanced performed similarly with higher mean results than ChatGPT-3.5, as shown in Table 3.

TNM staging

No application could provide the correct information in any case (n=0). The most common error was unsampled lymph nodes or unknown metastases staged as N0M0 (instead of NxMx). The application did not pick up hints in the clinical scenario that would be helpful in staging, e.g., in case 20, acellular mucin involving the subserosa was incorrectly staged as carcinoma in situ (Tis).

References

All three applications cited references that do not exist ("hallucinations") with 25-50% not being valid references, as shown in Table 3.

## Discussion

ChatGPT operates on OpenAI's GPT LLM framework, whereas Gemini is built upon Google's PaLM and LaMDA LLM families. A significant distinction between ChatGPT and Gemini is their training methodologies. ChatGPT versions 3.5 and 4.0 have been trained on predetermined datasets, last updated in January 2022 and April 2023, respectively. In contrast, Gemini Ultra boasts the largest training dataset (1.6 trillion parameters and 1.56 trillion words) and uniquely features the capability to access and retrieve information from the internet in real time [14].

Numerous studies showed that ChatGPT attained passing scores in the medical licensing exams of the United States [15], various American board examinations [16,17], and various international examinations [18,19]. However, there are only rare studies evaluating the performance of Gemini (previously called Bard), with the application doing exceptionally well in some examinations (orthopedic postgraduate examination questions) [20] and faring poorly in others (FRC ophthalmology practice questions and the national Indian premedical examination) [21,22].

In clinical medicine, each case comes with its unique challenges and tests the physician's in-depth medical knowledge and ability to think and reason. It is important to evaluate the accuracy of ChatGPT and Gemini in real-world clinical situations. A study by Kanjee et al. showed that ChatGPT-4.0 could give the correct D/D in 64% of the New England Journal of Medicine (NEJM) clinicopathological conferences [23]. 

There have been various studies evaluating ChatGPT in various realms of gastroenterology. Lahat et al. and Lim et al. evaluated its utility in answering patient questions in real-life situations related to gastroenterology, endoscopy, and inflammatory bowel disease. All studies highlight the weaknesses in these models and the need to make the tool better for practical use [7,8]. In other studies, even though ChatGPT was unsuccessful in passing the gastroenterology board examinations [11], it showed potential in assisting healthcare providers with the treatment of patients with GERD and the management of patients following colonoscopy, with definite limitations [9,10]. There is also potential use of ChatGPT in generating research questions of interest in gastroenterology as highlighted by Lahat et al. [7]. Laohawetwanit et al. studied the role of ChatGPT-4.0 in the classification of colorectal adenomas based on histopathological images and showed its low sensitivity, with the acknowledgment of its diagnostic limitations [24]. Wang et al. demonstrated that ChatGPT can deliver responses to pathology questions on par with, or superior to, those of trained pathologists, suggesting its potential as a valuable aid in pathology practice and resident education [25]. Cecchini et al. highlighted the contribution of LLMs to pathology education and advocated for a collaborative framework between humans and these models to advance pathology training [26]. Ours is the first study to evaluate the efficiency of three AI models in the realm of GI pathology.

Overall, our study provides a comprehensive assessment of ChatGPT-4.0, ChatGPT-3.5, and Gemini Advanced to evaluate a given clinicopathologic scenario in GI pathology. Our findings will hopefully provide insights to pathologists, gastroenterologists, and patients about the reliability, limitations, and shortcomings of these models.

The AI models' ability to "think and reason" was evaluated based on the scoring of the D/D. The 3.5 version did exceptionally well for certain entities (case 1) while being fairly poor on others (case 5). This could be because it is a constantly evolving tool and previous user experiences may cause that bias. Guastafierro et al. also reported similar results with ChatGPT faring exceptionally well in some clinical situations like SPN, pilomatrixoma, and sarcoidosis with poor performance in others [27].

Interpreting IHC stains and performing testing for prognostic and therapeutic significance is an essential component of the practice of pathology, and some interesting points were noted. The Ki67 proliferation index was commonly recommended in tumors like colonic adenocarcinoma and squamous cell carcinoma which does not have clinical utility. There was no mention of newer markers like INSM1 often used in clinical practice. Statements like "Desmin can differentiate between leiomyoma and leiomyosarcoma" by ChatGPT-3.5 are incorrect and highly misleading. Some strengths were identified as correctly identifying focal cytokeratin positivity in neuroendocrine tumors. Initially, ChatGPT-4.0 diagnosed case 18 as a solitary fibrous tumor of the stomach and recommended STAT6. When prompted that STAT6 is negative, it correctly diagnosed the case as inflammatory fibroid polyp. However, due to the confident tone of the diagnosis, it leaves additional testing to user discretion.

The final explanation provided by all three AI models was at best average and could be perceived as a disjointed thought process. Terms like neuroendocrine tumor and neuroendocrine carcinoma were used interchangeably which has marked therapeutic and prognostic significance. For TNM staging, in cases like colon cancer, it could identify the T stage correctly, but in conceptual matters (e.g., acellular mucin in low-grade appendiceal mucinous neoplasm (LAMN) upgrading the TNM stage), all AI applications fared poorly. These subtleties suggest that the application works robotically and is not yet developed enough to pick up the nuances of pathologic decision-making.

Almost all mean scores for ChatGPT-4.0 and Gemini Advanced ranged between 3.0 and 3.5, which is best classified as average. ChatGPT-3.5 showed the lowest performance levels in all four spheres showing a marked improvement in the data input and the training dataset for the 4.0 version and Gemini Advanced, though it still needs a lot to be desired. Explanation review brings forward the lack of understanding of the subject matter by putting random information together without being able to think it through. The practice of pathology requires analytical thinking which we are yet to see in these models.

Gemini Advanced repeatedly highlighted that the patient's history, imaging findings, and other clinical features guide the diagnosis with "Clinical correlation is the key", which is very essential for good pathology practice. Also, Gemini Advanced could look up the internet in real time and provide uniform resource locators (URL) for literature, which was an extremely helpful feature.

"Hallucinations", especially while citing references, make it an inefficient tool for scientific writing in its current form, as was previously noted [28]. All three chatbots cannot identify when they are providing misinformation and have a "highly confident language" that could be misleading. There have been studies in which ChatGPT and Gemini showed the ability to consistently generate health disinformation across various topics in a persuasive language and tone [2].

In their current forms, ChatGPT and Gemini cannot be used for pathology training, as they require histopathology knowledge to identify and input specific features to come to a diagnosis. For trainees, inaccurate inputs could lead them down an entirely different path. Also, with patients receiving their pathology reports, inputting that information in these applications may bring up a whole new diagnosis, which may lead to significant patient distress and mistrust of their diagnosis. ChatGPT-3.5, which is the free version, performed poorly in all spheres, and its universal free availability raises concerns.

The AI applications are not objective as the output is heavily dependent on their training dataset [1]. In healthcare, there is an absolute necessity for information to be accurate, unbiased, and latest, which is not the case currently. Studies have found that ChatGPT and Gemini models are currently inferior to human physicians and need human supervision [7,29,30]. Also, as inputted data is stored in these programs, patient privacy and maintenance of the Health Insurance Portability and Accountability Act (HIPAA) regulations need to be thoroughly addressed.

The strengths of ChatGPT-4.0 and Gemini Advanced were that they could come up with a fair D/D in cases of unknowns and recommend an initial set of stains in the diagnostic process. Even though the applications could modify the diagnosis based on additional prompting, this reinforces the need for human intervention to arrive at a correct diagnosis.

The limitations of our study were interobserver variability, and we used the scoring system to keep it uniform. Patient privacy was a limitation, and we used hypothetical clinical scenarios for our study. The ability to input real patient data will provide a better understanding of how these models work. However, as these applications store patient data, that is not an option at this point. The applications are not objective as the output is heavily dependent on their training dataset which could be biased. We have a small sample size and need additional studies to study how these models work in larger samples and various organ systems along with how histopathology images are evaluated which are key in coming to a final diagnosis.

## Conclusions

The latest ChatGPT-4.0 performs better than its predecessor ChatGPT-3.5, while Gemini Advanced shows great promise and shows understanding and clarity while dealing with the subject matter. These developments are exciting and will result in a transformation of the domain of AI language models each with its unique strengths and weaknesses.

ChatGPT-4.0 and Gemini Advanced could serve as adjunctive tools in pathology practice while exercising great caution. Currently, these applications cannot be used clinically and need human supervision. Once we have better control over the ethical considerations, it would be worthwhile to input patient data and evaluate the performance of these models. Both ChatGPT-4.0 and Gemini Advanced show potential as tools in clinical medicine, though continuous research and improvements are needed before they can be efficiently used in the medical domain.

## Figures and Tables

**Figure 5:**
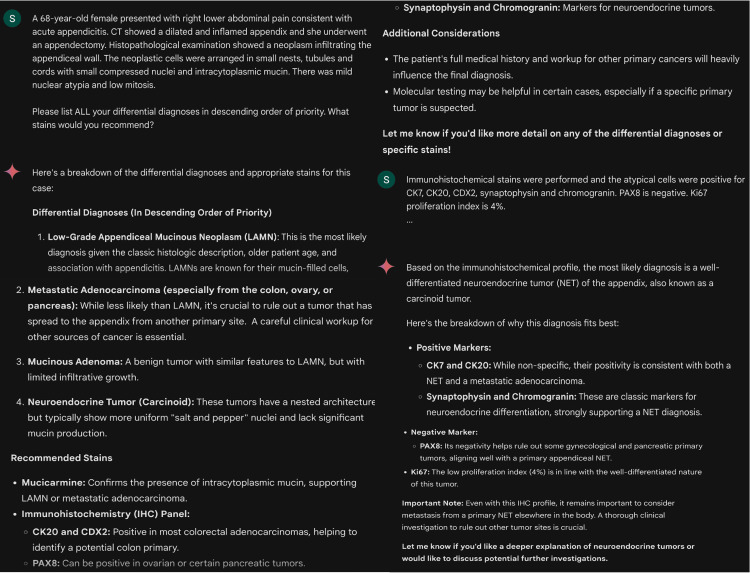
Sample screenshot of answer provided by Gemini Advanced for case 15

**Figure 6:**
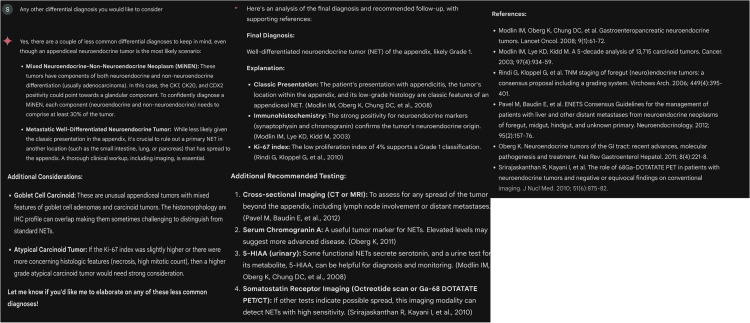
Sample screenshot of answer provided by Gemini Advanced for case 15 (continued)

**Figure 7:**
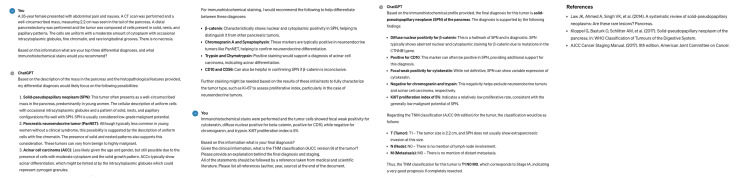
Sample screenshot of answer provided by ChatGPT-4.0 for case 2

**Figure 8:**
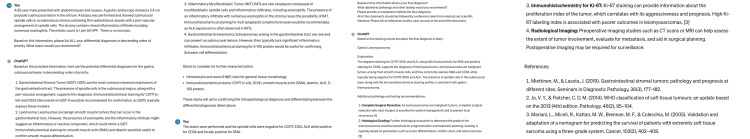
Sample screenshot of answer provided by ChatGPT-3.5 for case 18

## Appendices

Appendix A

**Table 5:** Twenty clinical case scenarios provided in our study CT: computed tomography; HPF: high-power field

Case no.	Diagnosis	Clinical scenarios
1	Well-differentiated neuroendocrine tumor of the pancreas, grade 1	A 50-year-old female patient presented with abdominal pain and diarrhea. A CT scan was performed, and a well-circumscribed mass, measuring 1.2 cm, was seen in the tail of the pancreas. A distal pancreatectomy was performed, and the tumor was composed of cells in nest and trabecular patterns. The cells are monotonous with finely granular cytoplasm and coarse-clumped chromatin. The mitotic count is 2 per 2 mm², and there is no necrosis. Two lymph nodes were identified which were negative for tumor.
2	Solid pseudopapillary neoplasm	A 35-year-old female patient presented with abdominal pain and nausea. A CT scan was performed, and a well-circumscribed mass, measuring 2.2 cm, was seen in the tail of the pancreas. A distal pancreatectomy was performed, and the tumor was composed of cells present in solid, nest, and papillary patterns. The cells are uniform with a moderate amount of cytoplasm with occasional intracytoplasmic globules, fine chromatin, and rare longitudinal grooves. There is no necrosis.
3	Pancreatic ductal adenocarcinoma	A 65-year-old male patient presented with weight loss and jaundice. A CT scan showed a mass, measuring 2.4 cm in the head of the pancreas. A Whipple procedure was performed, and the tumor was composed of atypical cells forming glands, with some cytoplasmic mucin within a desmoplastic stroma. Perineural invasion is present. Ten lymph nodes were present and were benign.
4	Mucinous cystic neoplasm of the pancreas	A 55-year-old female patient presented with abdominal pain. A CT scan showed a cystic lesion in the body of the pancreas, 8.5 cm, without communication to the main pancreatic duct. A Whipple procedure was performed, and the pathology showed a cyst lined by gastric foveolar epithelium with low-grade cytologic atypia, with underlying ovarian stroma. Three lymph nodes were present and were benign.
5	Granular cell tumor	A 60-year-old female patient presented with obstruction while swallowing. An esophageal endoscopy showed a sessile, yellowish, intact epithelium. A biopsy was done from the area which showed sheets of uniform round to polygonal cells with eosinophilic granular cytoplasm and central round vesicular nuclei. There was no mitosis or necrosis.
6	Colonic adenocarcinoma	A 70-year-old male patient presented with altered bowel habits. A colonoscopy showed an obstructive ulcer in the sigmoid colon, with a diagnosis of at-least intramucosal carcinoma. A left hemicolectomy was done, and the pathology showed a lesion with atypical cells predominantly forming glands invading through the muscularis propria into the pericolorectal tissues. The tumor is surrounded by a brisk inflammatory infiltrate. Fifteen lymph nodes were examined, of which four were positive.
7	Gastric signet ring carcinoma	A 45-year-old male patient presented with nausea and indigestion. An endoscopy showed a gastric ulcer with irregular borders, and a biopsy was highly suspicious of malignancy. A partial gastrectomy was done, and the pathology showed atypical single cells with cytoplasmic mucin and an eccentrically placed nucleus invading through the muscularis propria and involving the serosa. No gland formation was seen. Sixteen lymph nodes were examined, of which six were positive.
8	Gastrointestinal stromal tumor, spindle cell type, low risk of progression	A 65-year-old male patient presented with abdominal pain and occasional gastrointestinal bleeding. A CT scan was performed, and a solid mass, measuring 2.8 cm, was seen in the wall of the stomach. A gastrectomy was performed, and the tumor was composed of spindle cells in a palisading pattern with mild cytologic atypia and paranuclear vacuoles. The background shows mild mixed inflammatory infiltrate, and the mitotic count is 2 in 50 HPF. There is no necrosis.
9	Esophageal squamous cell carcinoma	A 60-year-old male patient presented with dysphagia. A CT scan showed a structuring of the esophageal wall in the middle third, measuring 2.4, with superficial ulceration. A biopsy was performed, and it showed ulcerated mucosa with a lesion composed of atypical cells with high-grade cytologic atypia in nests involving the submucosa. Areas of necrosis and brisk mitosis are identified.
10	Large cell neuroendocrine carcinoma of the rectum	A 60-year-old male patient presented with gastrointestinal bleeding and weight loss. A CT scan showed a necrotic mass in the rectum, 3.2 cm. A biopsy was performed and showed a lesion composed of atypical cells in nests, organoid pattern, and sheets. There was marked nuclear pleomorphism, with prominent nucleoli, extensive necrosis, and brisk mitoses.
11	Mucosa-associated lymphoid tissue lymphoma of the stomach	A 70-year-old male patient presented with nausea and epigastric pain. Endoscopy showed a discolored gastric mucosa with erosions. A biopsy was done, which showed a dense lymphoplasmacytic infiltrate destroying gastric glands. The lymphocytes are small and dys-cohesive and present in sheets with irregular nuclear contours and inconspicuous nucleoli with focal necrosis. The background stomach shows chronic active gastritis.
12	Primary extramammary Paget disease	A 65-year-old male patient presented with perianal itching and bleeding. On examination, an erythematous area was noted over the perianal area, and sigmoidoscopy showed rectal ulcers. A biopsy of the perianal area showed large, atypical cells in the basal epidermis, arranged singly and in nests containing cytoplasmic mucin. The cells had prominent nucleoli with occasional mitosis. The squamous mucosa showed reactive hyperplasia.
13	Malignant melanoma of the rectum	A 65-year-old male patient presented with bleeding per rectum and weight loss. On endoscopy, rectal ulcers with adjacent friable mucosa were noted. A biopsy was done which showed ulcerated mucosa with large, epithelioid cells with eosinophilic cytoplasm. There was marked nuclear pleomorphism with prominent nucleoli. Mitosis was brisk, and necrosis was noted.
14	Peritoneal malignant mesothelioma, epithelioid type	A 65-year-old female patient presented with weight loss in the past six months. She has a history of the removal of a 2 mm sigmoid colon polyp five years ago which showed a tubular adenoma. A CT scan was performed which showed multiple peritoneal nodules and peritoneal carcinomatosis which was biopsied. A peritoneal biopsy was performed which showed atypical cells involving the fibrous tissue. The cells were arranged in nests, sheets, and acinar-like structures, with an epithelioid morphology with coarse chromatin and occasional prominent nucleoli.
15	Goblet cell adenocarcinoma of the appendix, low grade	A 68-year-old female patient presented with right lower abdominal pain consistent with acute appendicitis. CT showed a dilated and inflamed appendix, and she underwent an appendectomy. Histopathological examination showed a neoplasm infiltrating the appendiceal wall into the muscularis propria. The neoplastic cells were arranged in small nests, tubules, and cords with small compressed nuclei and intracytoplasmic mucin. There were mild nuclear atypia and low mitosis.
16	Secondary Paget disease involving the rectum	A 65-year-old male patient presented with perianal itching and bleeding. On examination, an erythematous area was noted over the perianal area, and sigmoidoscopy showed rectal ulcers. A biopsy of the perianal area showed large, atypical cells in the basal epidermis, arranged singly and in nests containing cytoplasmic mucin and vesicular nuclei. The squamous mucosa showed reactive hyperplasia. Some atypical cells were seen in the submucosa.
17	Tubular adenoma with high-grade dysplasia	A 55-year-old male patient presented with bleeding per rectum. On endoscopy, there was a 12 mm polyp in the sigmoid colon which was completely excised. The histopathology showed normal colonic mucosa with an abrupt transition to the epithelium with crypts showing pseudostratification and elongated penicillate nuclei occupying the basal half of the cytoplasm with loss of cytoplasmic mucin. Focal areas showed crypts with cribriform architecture with increased nuclear/cytoplasmic ratio, nuclear rounding, open chromatin, and prominent nucleoli. These areas were limited to the epithelium.
18	Inflammatory fibroid polyp of the stomach	A 62-year-old male patient presented with abdominal pain and nausea. A gastric endoscopy showed a 2.4 cm polypoid submucosal lesion in the antrum. A biopsy was performed and showed submucosal spindle cells in an edematous stroma containing thin-walled blood vessels with a peri-vascular arrangement of spindle cells. The stroma contains mixed inflammatory infiltrates including numerous eosinophils. The mitotic count is 1 per 50 HPF. There is no necrosis.
19	Metastatic neuroendocrine tumor involving the liver	A 65-year-old female patient presented with abdominal pain and weight loss. On the CT scan, multiple liver lesions, the largest measuring 2.4 cm, were noted. A biopsy was done and showed medium-sized cells in nests and glands involving the hepatic parenchyma. The cells were monotonous with eosinophilic cytoplasm, fine chromatin, and inconspicuous nucleoli. Brisk mitosis and necrosis were absent.
20	Low-grade appendiceal mucinous neoplasm	A 65-year-old male patient presented with acute right lower abdominal pain consistent with acute appendicitis. A CT scan showed a dilated appendix with thickening of the appendiceal wall. An appendectomy was done which showed an appendix lined by a villous proliferation of the epithelium showing abundant apical mucin with elongated, penicillate nuclei and low-grade nuclear atypia. There were associated crypt loss and effacement of the muscularis mucosa. Acellular mucin was seen involving the subserosa.

Appendix B

Sample screenshots of answers provided by Gemini Advanced (Figure 5 and Figure 6), ChatGPT-4.0 (Figure 7), and ChatGPT-3.5 (Figure 8).
